# Supplementary material for: No Significant Association Between Vitamin C Supplements and Frailty in Korean Older Adults: A Cross-Sectional Analysis of the 2018–2019 Korea National Health and Nutrition Examination Survey
Source: Nutrients. 2025 Sep 17;17(18):2977. doi: 10.3390/nu17182977 (PMC12472814; doi:10.3390/nu17182977)
Supplement: Supplementary file 1 [file nutrients-17-02977-s001.zip › nutrients-3828582-supplementary.pdf]

Supplementary Table S1. Association of Vitamin C supplements use and frailty components.

	Frailty	Weight loss	Exhaustion	Weakness	Slowness	Low physical activity
Adjusted Probability % (95% CI)						
Non-DS	16.2 (14.1 - 18.3)	15.8 (13.3 - 18.2)	3.6 (2.5 - 4.6)	25.2 (22.4 - 27.9)	33.8 (31.1 - 36.5)	66.2 (63.2 - 69.2)
VIT C	11.6 (2.8 - 20.3)	8.6 (1.9 - 15.3)	0.0	15.4 (6.7 – 24.0)	31.2 (20.3 - 42.2)	59.7 (47.7 - 71.7)
Adjusted OR (95% CI)						
Non-DS	1	1	1	1	1	1
VIT C	0.65 (0.26 - 1.66)	0.49 (0.20 - 1.20)	NA	0.48 (0.22 - 1.05)	0.87 (0.49 -1.56)	0.74 (0.42 - 1.27)

Abbreviation: DS, dietary supplements; NA, non-available. Association between vitamin C supplement use and individual frailty components (weight loss, exhaustion, weakness, slowness, and low physical activity) among Korean adults aged ≥65 years, KNHANES 2018–2019. Odds ratios (ORs) and 95% confidence intervals (CIs) were estimated using multivariable logistic regression models adjusted for age, sex, education, income, body mass index, smoking, alcohol intake, chronic diseases, and protein intake.

Supplementary Table S2. STROBE Statement—checklist of items that should be included in reports of observational studies

	Item No.	Recommendation	Page No.	Relevant text from manuscript
**Title and abstract**	1	(*a*) Indicate the study's design with a commonly used term in the title or the abstract	Page 1	No Significant Association between Vitamin C Supplements and Frailty in Korean Older Adults: A Cross-Sectional Analysis of the 2018–2019 Korea National Health and Nutrition Examination Survey
		(*b*) Provide in the abstract an informative and balanced summary of what was done and what was found	Page 1	Abstract includes background, methods, exposure groups, results, and conclusions
**Introduction**				
Background/rationale	2	Explain the scientific background and rationale for the investigation being reported	Page 1-2	Section: Introduction.
Objectives	3	State specific objectives, including any prespecified hypotheses	Page 2	Section: Introduction (end)
**Methods**				
Study design	4	Present key elements of study design early in the paper	Page 2	Section: Methods, Study Design
Setting	5	Describe the setting, locations, and relevant dates, including periods of recruitment, exposure, follow-up, and data collection	Page 2-3	KNHANES 2018–2019, nationwide representative survey of Korea
Participants	6	(*a*) Give the eligibility criteria, and the sources and methods of selection of participants	Page 2-3	Sections: Methods, Study Population
Variables	7	Clearly define all outcomes, exposures, predictors, potential confounders, and effect modifiers. Give diagnostic criteria, if applicable	Page 2-6	Sections: Methods, Frailty Definition, vitamin C Intake, Covariates
Data sources/ measurement	8*	For each variable of interest, give sources of data and details of methods of assessment (measurement). Describe comparability of assessment methods if there is more than one group	Page 2-6	Sections: Materials and Methods (KNHANES 2018–2019)

Bias	9	Describe any efforts to address potential sources of bias	Page 4-5<br>Page 9	Addressed through covariate adjustment, comparator group design (vitamin C only vs. other supplements vs. non-users). Section: Discussion (Limitations)
Study size	10	Explain how the study size was arrived at	Page 2-3, Figure 1	All eligible older adults from 2018 to 2019 KNHANES were included; no formal sample size calculation.
Quantitative variables	11	Explain how quantitative variables were handled in the analyses. If applicable, describe which groupings were chosen and why	Page 5	Vitamin C intake categorized as <100, 100–999, 1000–1999, ≥2000 mg/day; justified by RNI/UL thresholds.
Statistical methods	12	(*a*) Describe all statistical methods, including those used to control for confounding	Page 5-6	Survey-weighted logistic regression models were estimated using STATA, adjusting for sociodemographic, lifestyle, and nutritional covariates
		(*b*) Describe any methods used to examine subgroups and interactions	Page 7	Interaction terms tested in logistic models Section: Table 3, Supplementary Tables S1
		(*c*) Explain how missing data were addressed	Page 2	Key variable missing excluded Section: Methods, Statistical Analysis
		(*d*) If applicable, describe analytical methods taking account of sampling strategy	Page 5-6	Complex survey design of KNHANES using kstrata, PSU, and the combined weight variable

		(*e*) Describe any sensitivity analyses	Page 4	Section: Methods, Statistical Analysis
**Results**				
Participants	13*	(a) Report numbers of individuals at each stage of study—e.g., numbers potentially eligible, examined for eligibility, confirmed eligible, included in the study, completing follow-up, and analysed	Page 2-3, Page 6	Figure1
		(b) Give reasons for non-participation at each stage	Page 3	national survey design
		(c) Consider use of a flow diagram	Page 3	Figure1
Descriptive data	14*	(a) Give characteristics of study participants (e.g., demographic, clinical, social) and information on exposures and potential confounders	Page 6	Table 1
		(b) Indicate number of participants with missing data for each variable of interest	Page 2-3, Page 6	Figure1, Table 2-3
Outcome data	15*	Report numbers of outcome events or summary measures	Page 6-8	Sections: results, Table 2-4
Main results	16	(*a*) Give unadjusted estimates and, if applicable, confounder-adjusted estimates and their precision (e.g., 95% confidence interval). Make clear which confounders were adjusted for and why they were included	Page 7-8	Adjusted ORs presented; predicted probabilities Table 3
		(*b*) Report category boundaries when continuous variables were categorized	Page 7-8	Table 4
		(*c*) If relevant, consider translating estimates of relative risk into absolute risk for a meaningful time period	Page 7	absolute risk differences reported Results, Table 3
Other analyses	17	Report other analyses performed—e.g., analyses of subgroups and interactions, and sensitivity analyses	Page 8,13	Supplementary Table S1: frailty components
**Discussion**				
Key results	18	Summarise key results with reference to study objectives	Page 8	Sections: Discussion para 1
Limitations	19	Discuss limitations of the study, taking into account sources of potential bias or imprecision. Discuss both direction and magnitude of any potential bias	Page 9-10	Cross-sectional design, reverse causation, recall bias, measurement error, residual confounding, healthy-user bias Discussion (limitations)
Interpretation	20	Give a cautious overall interpretation of results considering objectives, limitations, multiplicity of analyses, results from similar studies, and other relevant evidence	Page 8-10	Discussion (overall)

Generalisability	21	Discuss the generalisability (external validity) of the study results	Page 10	Discussion (Strengths and generalisability)
**Other information**				
Funding	22	Give the source of funding and the role of the funders for the present study and, if applicable, for the original study on which the present article is based	Page 10	No funding

*Give information separately for cases and controls in case–control studies and, if applicable, for exposed and unexposed groups in cohort and cross-sectional studies.

Note: An Explanation and Elaboration article discusses each checklist item and gives methodological background and published examples of transparent reporting. The STROBE checklist is best used in conjunction with this article (freely available on the Web sites of PLoS Medicine at http://www.plosmedicine.org/, Annals of Internal Medicine at http://www.annals.org/, and Epidemiology at http://www.epidem.com/). Information on the STROBE Initiative is available at www.strobe-statement.org.
